# The Magnetization of a Composite Based on Reduced Graphene Oxide and Polystyrene

**DOI:** 10.3390/nano11020403

**Published:** 2021-02-05

**Authors:** Alexander N. Ionov, Mikhail P. Volkov, Marianna N. Nikolaeva, Ruslan Y. Smyslov, Alexander N. Bugrov

**Affiliations:** 1 Ioffe Institute, Politekhnicheskaya 26, 194021 St. Petersburg, Russia; ionov@tuch.ioffe.ru (A.N.I.); m.volkov@mail.ioffe.ru (M.P.V.); 2 Institute of Macromolecular Compounds, Russian Academy of Sciences, Bolshoy pr-t 31, 199004 St. Petersburg, Russia; marianna_n@mail.ru (M.N.N.); urs@macro.ru (R.Y.S.); 3 Institute of Biomedical Systems and Biotechnology, Graduate School of Biomedical Systems and Technology, Peter the Great St. Petersburg Polytechnic University (SPbPU), Polytechnicheskaya 29, 195251 St. Petersburg, Russia; 4 Department of Physical Chemistry, Saint Petersburg Electrotechnical University (ETU “LETI”), ul. Professora Popova 5, 197376 St. Petersburg, Russia

**Keywords:** graphene-based materials, defective graphene nanosheets, magnetic properties, superconductivity, magnetoresistance

## Abstract

The use of reduced graphene oxide (r-GO) is a promising way of fabricating organic–inorganic composites with unique electrical and magnetic properties. In our work, polystyrene/r-GO composites were synthesized, in which both the components are linked together by covalent bonds. The r-GO used differs from the graphene obtained from graphite through mechanical exfoliation using the ‘scotch tape’ by presenting many structural defects. Binding in the composite structure between the components was confirmed by infrared spectroscopy. Elemental analysis was carried out by energy dispersive X-ray spectroscopy. Scanning electron microscopy, X-ray diffraction, and Raman spectroscopy were used to monitor the 2D-order in exfoliated r-GO galleries. Using a vibrating-sample magnetometer, we have shown that the composite magnetization loops demonstrate type-II superconductivity up to room temperature due to r-GO flakes. We believe that a strain field in the r-GO flakes covalently binding to a polymeric matrix is responsible for the superconductivity phenomena.

## 1. Introduction

Since discovering the effect of superconductivity, researchers worldwide have focused on creating superconducting materials with a high critical temperature. Back in 1974, researcher K. Antonovich published an article stating that the current through the junction of amorphous graphite strongly depends on a small external magnetic field, as predicted by Josephson for two weakly coupled superconductors [1]. The effect existed even at room temperature.

Since 1980, researchers started the race to get organic superconductivity materials. First, superconductivity was observed by resistive measurements in the quasi-one dimensional organic conductor di-(tetramethyltetraselenafulvalene)-hexafluorophosphate, (TMTSF)2PF6 under a hydrostatic pressure of 12 kbar with a transition temperature of 0.9 K [2]. In 2020, Elliot Snider with coworkers approached room-temperature superconductivity by proposing the pressure-driven disproportionation of hydrogen sulfide (H_2_S) to H_3_S, with a confirmed transition temperature of 203 K at 155 GPa [3]. As for bilayer graphene, a two-dimensional allotrope of carbon, in 2018 it was discovered to be a superconductor when layers were twisted on the ‘magic’ angle of about 1.1° [4]. Theoretically, it was clarified that the effective d + id pairing interaction strongly increases as the on-site Coulomb interaction increases, indicating that the superconductivity is driven by electron–electron correlation [5].

One paper [6] demonstrated that the superconductivity of graphite–sulfur composites occurs within some sort of “grains” or domains. In particular, magnetization measured at *T* = 7 K showed a characteristic type II superconductor hysteresis loop. High-resolution magnetoresistance data in highly oriented pyrolytic graphite (HOPG) thin samples manifest nonhomogenous superconductivity with a critical temperature of *T*_c_ = 25 K and higher [7]. It was found that the magnetization of pristine graphite powder, with a grain size of several tens of micrometers, after a simple treatment with pure water, shows clear and reproducible granular superconducting behavior with a critical temperature above 300 K [8]. The transport characteristics of thin lamellae with tens of two-dimensional interfaces between crystalline graphite regions reveal a Josephson-like behavior with zero resistance states at low enough temperatures and input currents [9]. In [10], the observation of persistent currents in the ring-shaped container, which did not decay for 50 days, suggests that the HOPG plates immersed in n-heptane and n-octane really entered a zero-resistance state at room temperature. The critical value of Tc at which the Josephson behavior sets depends on the interface width of carbon lamellae and vanishes for widths below 200 nm [11]. Evidence for type II superconductivity, based on measurements of transport current, magnetic susceptibility, Hall effect, and vortex state, was obtained in phosphorus-doped graphite and graphene at temperatures of about 260 K [12]. The trapped magnetic flux in the finely ground pyrolytic graphite, which has been observed on cooling of the sample from room temperature in a magnetic field, may reflect the presence in it of a granular high-temperature superconducting phase [13]. In [14], a magnetic field dependent electronic gap was observed, suggestive of local superconductivity, in the point-contact spectrum of microcrystalline graphite. Using the Bardeen–Cooper–Schrieffer (BCS) theory, the authors obtained a critical superconductivity temperature of 14 K. Unusual hysteresis features that point out to the possible presence of vortex states were observed in HOPG layers [15]. These observations may indicate the superconductive signals. In [16], one found the superconducting correlations in the local area of nanographite film surface—AC-to-DC conversion associated with the reversed Josephson effect, pinning of vortices on columnar topological structure observed in atomic force and magnetic force microscope, non-zero current at zero voltage in scanning tunneling microscope.

Almost all the authors indicated above obtained the results dealing with high-temperature superconductivity on HOPG. However, over many years of observing this effect, no answer has been received about why superconductivity arises in some HOPG samples. Meanwhile, there are many ones in which superconductivity cannot be detected, despite a similar diamagnetic response. Such poor reproducibility of experimental results has often caused skepticism about their reliability.

Graphene has focused materials-science researchers’ attention since it has many aspects of promising practical applications [17,18]. The range of applications for graphene can be expanded by combining it with other materials, thereby significantly changing its properties. This is achieved by chemically modifying the graphene surface with soft substances or solid inorganic materials [19]. Ma et al. carried out current-sensing atomic force microscopy (CSAFM) experiments for laser-reduced graphene oxide (r-GO) [20]. One revealed that after laser reduction, the high-conductivity r-GO spots appear on the graphene oxide surface in the form of two-dimensional torus shapes of several micrometers. In other work [21], the authors synthesized heavily-reduced GO films exhibiting ferromagnetic properties with a coercivity of 40 Oe and a saturation magnetization of 7.0 emu/g at 300 K. The researchers in [22] proposed that doped graphene can achieve an electronically mediated superconductivity provided that the chemical doping is near a van Hove singularity. It means that a divergence in the density of states (DOS) and the lattice symmetry is preserved. As a result, it has led to further research of fundamental properties in developing a new class of materials based on polymer/graphene composites [23,24]. The nanosheet galleries in the exfoliated graphite (EG) structure are easily intercalated with monomers or polymers. After intercalation, EG particles are distributed in the polymer matrix in the form of nanosheets with thicknesses from 10 to 50 nm. EG-containing nanocomposites based on polymethyl methacrylate (PMMA) [25], styrene-acrylonitrile copolymer [26], polystyrene (PS) [27], and PS-PMMA mixture [28] have been obtained to achieve electroconductive properties. As to our approach, the idea is to reach twisted multilayer graphene using covalently bonded macromolecules. We reported on a composite based on polystyrene (PS) with multilayer reduced graphene oxide (r-GO), which exhibited a Josephson type of current-voltage characteristic (CVC) up to room temperature [29,30]. A Josephson type of CVC indicates that superconductivity exists with a high critical temperature (*T*_c_), till a room one, in r-GO flakes linked by chemical bonds with PS chains.

In the following studies, the magnetization of the composite (*M*_comp_) for the same type of r-GO flakes was measured [31]. We have obtained confirmation of *M*_r-GO_(H) superconducting behavior up to room temperature after subtraction of *M*_comp_’s components associated with diamagnetic background slopes of r-GO flakes and PS, as well the paramagnetic contribution of some unknown impurities in a polymer matrix. However, such a subtraction procedure may cause critics to doubt the reliability of the result, because the small superconducting effect is concealed within a relatively larges diamagnetic and paramagnetic effects. Here, it should be noted that the magnetization curves showed hysteresis with huge coercivity (*H*_c_) at *M* = 0. If the magnetization curves were related to ferromagnetic order, then we could detect it. However, the electron spin resonance investigation did not demonstrate magnetic ordering in the composite [32], which once again confirms the superconducting behavior of *M*_r-GO_(H).

In this regard, we believe that further study of such artificially created structures with the participation of various forms of carbon filler and polymers, in which many more parameters can be controlled than in HOPG, will provide an answer to the puzzle of the superconductivity in carbon-based materials, and this will be important for practical application.

In this work, we investigated the effects of modifying chemically the multilayer graphene on the static magnetization of the r-GO-containing composite based on PS (Figure S1 in ESM). This paper shows that—(i) the composite magnetization plots can demonstrate type-II superconductivity without subtracting any diamagnetic and paramagnetic contributions; (ii) the effect of superconductivity depends on the oxygen-containing residues detected through a paramagnetic signal when measuring static magnetization at low temperature and linking r-GO flakes with polystyrene macromolecules.

## 2. Materials and Methods

### 2.1. Materials

The technical sequence for the synthesis of the two PS/r-GO composites in this work was as follows:

(1) As the first step from natural pristine graphite, we produced graphite oxide by a modified Hummers method. The initial graphite oxide was synthesized via oxidation of the natural crystalline graphite by potassium permanganate in sulfuric acid in the presence of sodium nitrate, using a procedure that was close to that described in [33]. During the oxidation, many different functional groups, such as hydroxy, epoxy, and carboxylic ones, attach to the basal planes and edges of the graphene sheets [34,35,36]. A significant property of graphite oxide is the ability to undergo complete exfoliation in water, yielding colloidal suspensions of individual graphite oxide nanosheets. The C/O ratio, obtained by X-ray photoelectron spectroscopy (XPS) analysis (the Russian–German synchrotron radiation beamline at bending-magnet D16-1A of the BESSY-II electron storage ring, Berlin, Germany [37]) for graphite oxide, is in a range of 4:1–2:1 [38,39]. Graphite oxide films were prepared on the surface of silicon substrates by drying aqueous suspension in air at 120 °C. Besides, when reducing in the Ar atmosphere according to the XPS and energy dispersive X-ray analysis (EDX) (Table 1, *cp* composites 1 and 2) [38,39], the C/O ratio 7.45 corresponds to a lower number of oxygen-containing groups than in H_2_ when this ratio is 5.82.

(2) As shown by the XPS study, high temperatures (*T* > 750 °C) are necessary to achieve good reduction of graphene oxide when the C/O ratio can be higher than 13 [40]. On annealing, part of O-containing functional groups is removed from the r-GO flakes, but simultaneously they leave defects in their place, in the form of vacancies, structural deviations, and distortion [41]. After heat treatment, the oxygen content decreases significantly in r-GO but, in many cases, does not entirely disappear [42]. When studying static magnetization, this can be indicated by the presence of a paramagnetic contribution at low temperatures. Thus, r-GO differs from graphene produced from graphite by mechanical exfoliation using the ‘scotch tape’ method, which lacks these defects [17].

(3) Before the synthesis of the r-GO-containing composites based on PS (Figure S1 in ESM), we carried out the functionalization of r-GO flake surfaces using 3-(trimethoxysilyl) propyl methacrylate (TMSPM) (CAS Number: 2530-85-0, Aldrich, St. Louis, MO 63103, USA) to improve the interaction with the polymer matrix [43,44]. For synthesizing composites 1 and 2, we used r-GO upon reduсing in an Ar (900 °C) or H_2_ atmosphere (900 °C), correspondingly (Table 1).

### 2.2. Methods of Characterization

Control over the organosilicon modifier’s attachment to the surface of two-dimensional r-GO nanostructures was carried out using FTIR spectroscopy on a Vertex 70 spectrometer (Bruker Optik GmbH, Ettlingen, Germany), which is equipped with an attenuated total reflectance device (Pike Technologies Inc., Madison, WI 53719, USA).

Raman spectra of r-GO reduced in argon and hydrogen have been obtained using a 532 nm laser with a power of 0.2 mW in the range of 80–3700 cm^−1^ Raman shifts in a focal field of 25 × 1000 μm (see ESM as well).

The elemental constituents of PS/r-GO composite and their components were measured using—(i) Auger electron and X-ray photoelectron spectroscopy measurements performed on a Multitechnique 5500 spectrometer from Physical Electronics (USA); (ii) a TESCAN VEGA 3 SBH scanning electron microscope (TESCAN, Brno, Czech Republic) with an INCA Energy 250/X-max 20 microanalysis system (Oxford Instruments Nanoanalysis Ltd., High Wycombe Bucks, UK).

The X-ray diffraction (XRD) analysis of the crystallinity size and the assessment of the number of layers for r-GO nanosheets was performed using a Rigaku SmartLab diffractometer (Rigaku Corporation, Tokyo, Japan) with Cu*K*_α_ radiation.

Magnetic measurements on the samples were performed in the temperature range of 2–400 K and magnetic fields from 0 to ±10 T. The composite was placed into a separate diamagnetic plastic holder with an inner diameter of about 3 mm and installed in a vibrating-sample magnetometer based on Quantum Design PPMS^®^ (Quantum Design Inc., San Diego, CA 92121, USA).

## 3. Results

### 3.1. Impurity Control

The chemical compositions were determined from the maximum possible area of the test samples. EDX analysis confirms the absence of 3d metal impurities in native or TMSPM-modified r-GO flakes and in those included in the composite structure (Table 1). In this case, the presence of an organosilicon compound responsible for the covalent binding of r-GO flakes to styrene macromolecules can be traced in the EDX spectra for composite 2 (Figure S2 in ESM). Auger electron and X-ray photoelectron spectroscopy measurements did not reveal the presence of magnetic impurities in the r-GO flakes and composites.

### 3.2. The Analysis of IR Spectra

**Figure 1:**
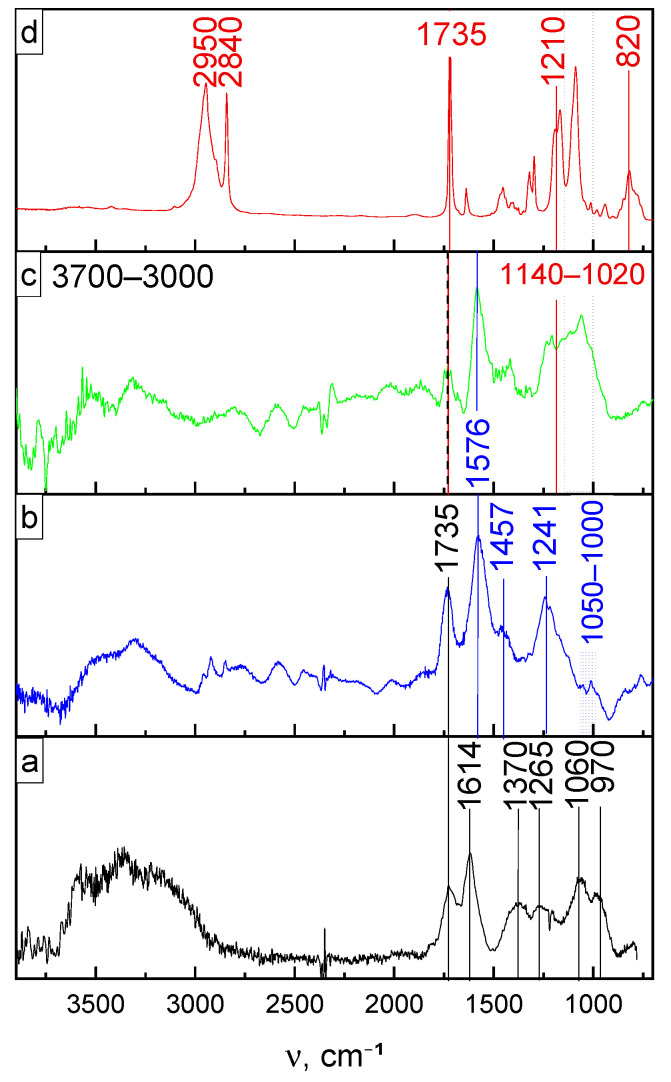
FTIR spectra of graphite oxide (**a**), r-GO (**b**), r-GO modified (**c**) with TMSPM (**d**). The r-GO was obtained in an H_2_ atmosphere.

The IR spectrum exhibited intense broad bands in the region of 3700–3000 cm^−1^ due to O–H stretching vibrations of carboxyl groups and adsorbed water for the initial graphite oxide. Besides, peaks centered at 1370 and 1265 cm^−1^ correspond to skeletal vibrations of C–OH, C–O–C bonds. The characteristic band maximum for stretching vibrations of C=O at 1735 cm^−1^ was also recorded. The remaining signals at 1060 and 1614 cm^−1^ can be attributed to the skeletal vibrations of C=C unoxidized sp^2^ C-bonds and C–O stretching vibrations of alkoxy groups, respectively (Figure 1a). The shoulder at 970 cm^−1^ is commonly associated with epoxyl, ether, and peroxide groups. In the spectrum of partially reduced graphene oxide in a hydrogen atmosphere, the bands’ intensity in the region of 3000–3700 cm^−1^ decreases, and three main peaks at 1735, 1576, and 1241 cm^−1^ are traced (Figure 1b). The band located at 1735 cm^−1^ is associated with the stretching vibrations of C=O in the residual carboxyl and carbonyl groups. Absorption in the 1576 and 1457 cm^−1^ is due to in-plane bending vibrations of C=C bonds. The last peak (1241 cm^−1^), with a hardly distinguishable shoulder in the range of 1000–1050 cm^−1^, corresponds to the stretching vibrations of C–O–C alkoxyl and epoxyl groups. After functionalization of r-GO flakes using the double bond modifier TMSPM, additional absorption bands appear in the FTIR spectrum (Figure 1c) in the frequency range of 1020–1140 cm^−1^, which corresponds to the stretching vibrations of the following bonds—Si–O, Si–OH, Si–O–C and Si–O–Si. In turn, the band of the methoxyl groups of the organic silicon compound, centered at 820 cm^−1^, disappears since they are hydrolyzed during the modification of the r-GO flakes (compare Figure 1c,d). The bands of ester groups on the r-GO surface and in the TMSPM structure overlap, and the vinyl bond signal appears at 1210 cm^−1^. This fact indirectly confirms the efficiency of the performed surface modification of the r-GO flakes.

**Figure 2:**
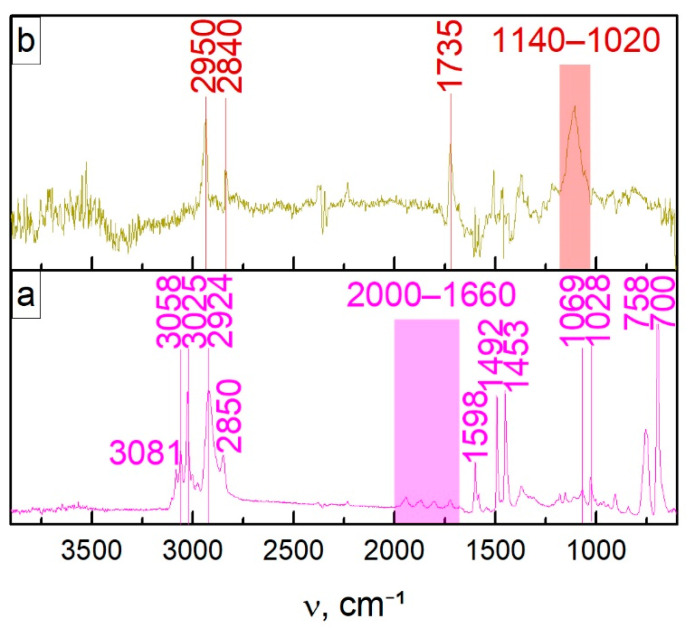
FTIR spectrum of composite 2 (**a**) and the difference between composite and PS matrix (**b**).

Styrene copolymerization with TMSPM on the r-GO flake surface was carried out according to [45], leading to a PS/r-GO composite. For the latter one, band maxima at 3081, 3058, 3025 cm^−1^ are observed in the FTIR spectrum, corresponding to the absorption of stretching vibrations of aromatic C–H bonds. The two peaks centered at 2924 and 2850 cm^−1^ are caused by asymmetric and symmetric stretching vibrations of CH_2_ groups. The peaks in the range of 1660–2000 cm^−1^ can be attributed to monosubstituted benzene rings. The spectrum contains vibrations of C=C bonds of aromatic rings at 1598, 1492, and 1453 cm^−1^. The bands of twisting vibrations of the C–H bonds in the phenyl ring plane (1069, 1028 cm^−1^) and outside it (700, 758 cm^−1^) are also visible. Along with the characteristic bands of PS (Figure 2a), the spectrum of its composite with r-GO contains signals of stretching vibrations of C=O (1735 cm^−1^) and silicon-containing groups (1020–1140 cm^−1^) (Figure 2a). Further, the polymer matrix spectrum was subtracted from the one of composites—PS with covalently bonded r-GO flakes (Figure 2b).

### 3.3. The Analysis of Raman Spectra

**Figure 3:**
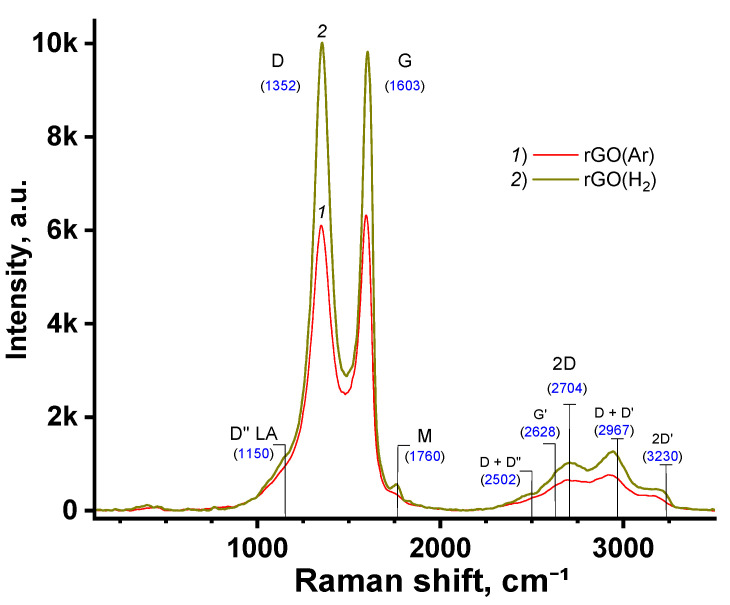
Raman spectra of rGO reduced in an Ar (1) or H_2_ (2) atmosphere. The main peaks are labelled according to Ferrari [46] and Ma [20].

In Figure 3, we compare the Raman spectra for r-GO(H_2_) with r-GO(Ar) reduced in molecular hydrogen and argon medium, respectively. Based on model ideas about the nature of the peaks [20,46] in the Raman curves, we deconvolved the spectra contour using the Voigt function in the OriginPro 2021 (version 9.8.0.200, OriginLab Corporation, Northampton, MA, USA) software (Figure S3 in ESM). The contours of the Raman spectra for the r-GO under investigation appear to be approximately the same.

In the Raman spectra (Figure 3), we observe the D, G, 2D peaks typical for the single-layer graphene for both types of the reduced carbon structures [46]. Besides, the shoulder D” at ca. 1100 cm^−1^, resulting from the acoustic longitudinal vibrations (LA) in the nanosheets consisting of several layers of defective graphene, few-layer graphene, is observed. This mode is associated with the *sp*²–*sp*³ edge bonds, activated in the disordered graphite lattice [47]. For r-GO obtained in an H_2_ atmosphere, the D” shoulder contribution is higher than when Ar was used, indicating that the structure is more defective in the case of H_2_. Also, in the shift range 2400–3300 cm^−1^, where the second-order peaks are located [20,46], the contribution of the sum D + D” of the two modes for r-GO(H_2_) can be seen. At 1765 cm^−1^, the M peak is attributed to an intravalley resonant second-order scattering process. This combinational mode arises based on out-of-plane breathing and in-plane longitudinal optical oscillations of the layer [20].

### 3.4. XRD Analysis of Initial and Surface Modified r-GO Flakes

**Figure 4:**
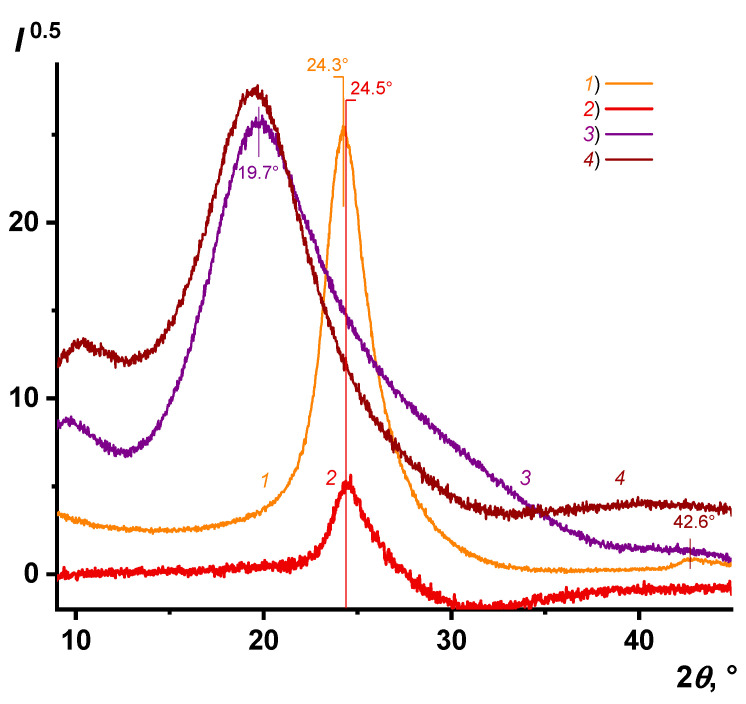
XRD patterns of initial r-GO(H_2_) flakes (*1*), surface modified ones (*2*), polystyrene (*3*), and composite 2 (*4*).

XRD patterns of both initial and modified TMSPM r-GO flakes have a characteristic peak at 24°, corresponding to the (002) graphite plane. Concerning this reflex, the Bragg equation was used to estimate the interplanar distance (*d*) between graphene layers. For determining the average height of the stacked layers (average crystalline size, *D_p_*), the calculation was carried out according to the Scherrer formula based on the value of the grazing angle and the full width at half maximum (FWHM) (Figure 4 and Table 2) [48,49].

According to the ratio of maximum intensities, *I*_D_ and *I*_G_, corresponding to vibrational modes D and G in the Raman spectra, we estimated the size of the cluster diameter or in-plane correlation length, $L_{a}$, following Tuinstra and Koenig [50]:

$$\frac{I_{D}}{I_{G}}=\frac{C\left(\lambda \right)}{L_{a}},$$

where *C*(*λ*) is ca. 4.4 nm, and *λ* is an excitation laser wavelength (514 nm). It turned out that the $L_{a}$ magnitude for r-GO reduced in an Ar or H_2_ atmosphere is approximately the same and is about 4.6 and 4.3 nm, correspondingly. So, if taking into consideration the XRD data obtained (Table 2), a crystalline domain represents a graphite nanosheet with an aspect ratio of ca. 1.3–1.6 and a thickness of ca. 8–9 layers.

In the XRD pattern, the peak’s position remains practically unchanged, and the FWHM values are comparable within the error limits for TMSPM-modified and initial r-GO flakes (Figure 4, curves *1* and *2*). At that, the interplanar distance is ca. 0.365 nm that more than 0.335 nm for pristine graphite. This indicates some defects in the structure of r-GO led to expanding graphite nanosheets [28].

Both the small amount of r-GO in the composite and the large contribution of short-range order recorded on the X-ray diffraction pattern of the unfilled polystyrene matrix do not allow one to judge the shifting of graphene layers and the expanding of nanosheets because of covalently binding graphite nanosheets with macromolecules (Figure 4, curves *3* and *4*).

**Figure 5:**
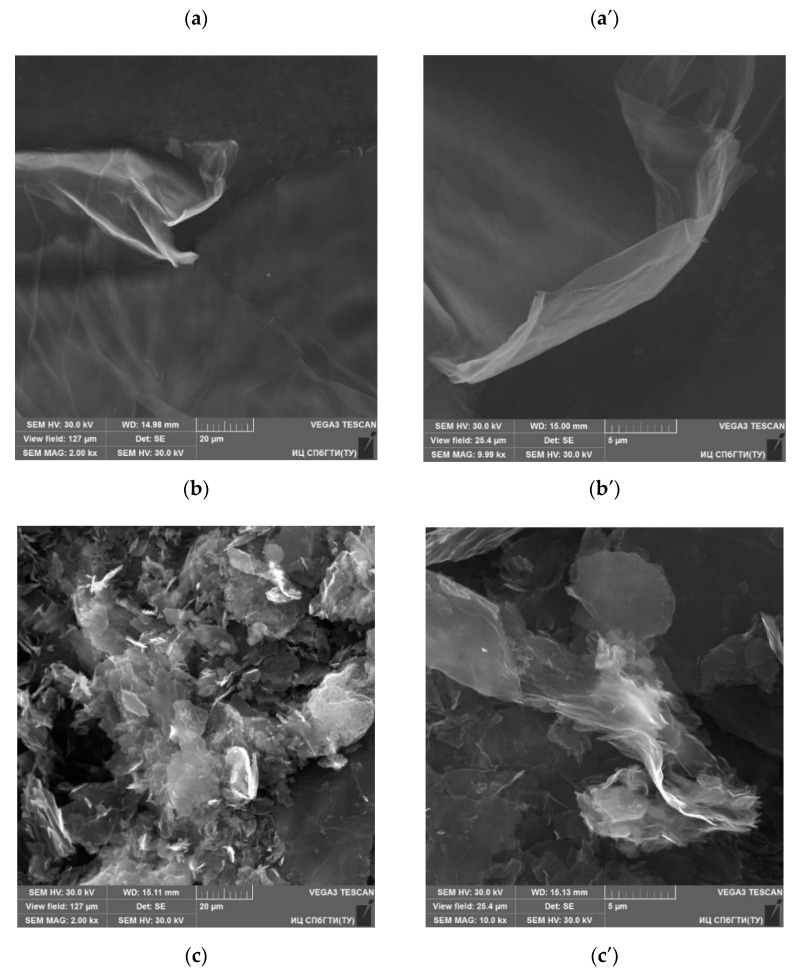

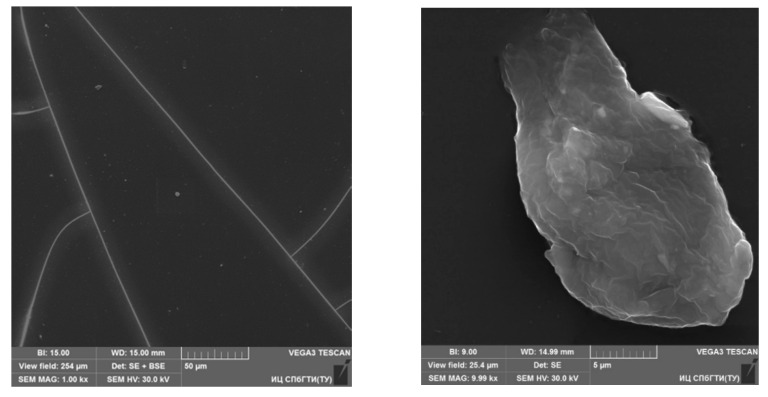
Scanning electron microscopy (SEM) micrographs of the initial (**a**,**a’**), surface modified r-GO flakes (**b**,**b’**) and a film surface for composite 2 (**c**,**c’**) taken at 1 k× (**c**), 2 k× (**a**,**b**) and 10 k× (**a’**,**b’**,**c’**) magnifications. The r-GO was obtained in an H_2_ atmosphere.

However, the ultrasonic dispersion of r-GO flakes with subsequent covalent attachment of TMSPM to their surface during the condensation reaction promoted significant refinement of two-dimensional carbon structures hundreds of microns in size (Figure 5a,a’) to plates with an average aspect ratio of about 8 × 13 µm (Figure 5b,b’), according to SEM micrographs. Inclusions of flakes of similar dimensions emerge on the formed PS/r-GO composite film’s surface (Figure 5c,c’).

### 3.5. The Analysis of Magnetization

**Figure 6:**
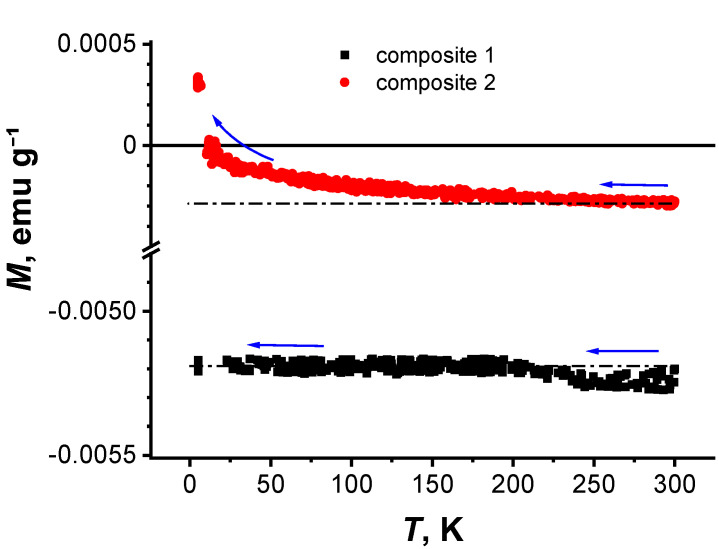
Temperature dependence of the magnetic moments for composites 1 and 2 in cooling at *H* = 500 Oe.

Figure 6 shows the magnetic moments of two composites 1 and 2 vs. temperature in cooling from 300 to 2 K in the field *H* of 500 Oe.

According to the EDX data when obtaining r-GO in H_2_, the C/O ratio, 5.82 corresponds to a higher number of oxygen-containing groups than those obtained in Ar, where this ratio is 7.45 (see Section 2.1). Moreover, having a greater contribution to the D″ peak for r-GO(H_2_) than r-GO(Ar) allows for more oxygen defects in the structure of rGO(H_2_) (see Section 3.3). As can be seen from Figure 6, indeed, composite 1 exhibits diamagnetic magnetization, which is independent of temperature. Whereas for composite 2, the magnetization behaves diamagnetically at high temperatures (see curve 2 in Figure 6). However, with decreasing temperature, the magnetization crosses the temperature axis and becomes positive. A gradually increasing paramagnetic response suppresses the diamagnetic behavior of the composite 2 due to the remaining patches of graphene oxide on the r-GO flake surfaces. On the contrary, paramagnetic growth at low temperatures is not observed in composite 1.

**Figure 7:**
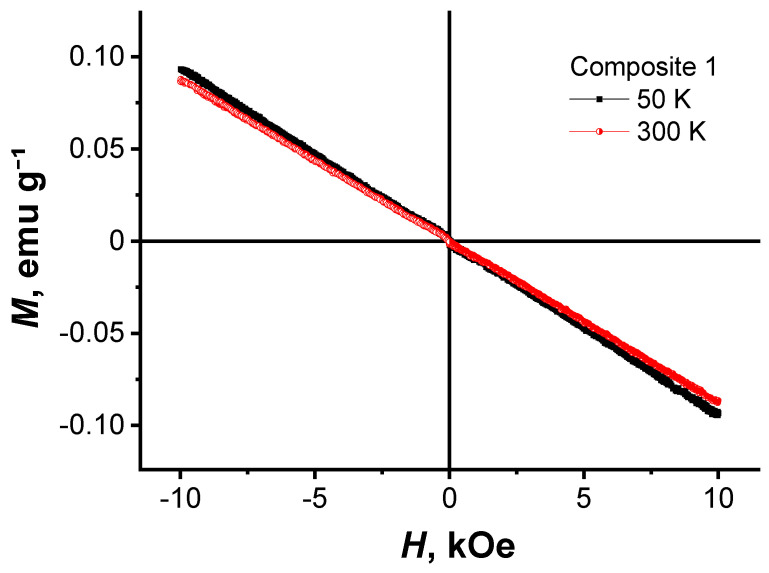
Dependence of the magnetic moment versus magnetic field for composite 1 at *T* = 50 and 300 K.

Figure 7 shows the field dependence of the static magnetization for composite 1 at *T* = 50 and 300 K. According to the obtained dependence *M*(*H*), composite 1 exhibits diamagnetic behavior in the entire magnetic field range.

**Figure 8:**
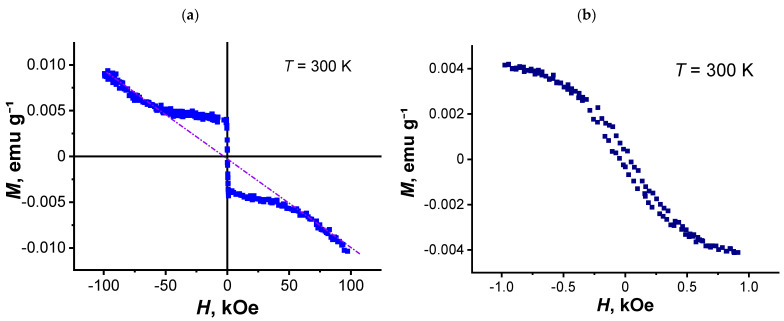
Dependence of the magnetic moment on the magnetic field at *T* = 300 K for composite 2 in strong (**a**) and weak (**b**) magnetic fields.

Figure 8a,b shows the field dependence of the static magnetization for the composite 2 at *T* = 300 K. In weak magnetic fields, an abnormally high increase in magnetization (a) with a small hysteresis (b) turned counterclockwise is observed. With an increase in the magnetic field, the anomalously high component of the magnetization gradually decreases, as is observed in superconductors [51,52,53,54]. Only diamagnetism is revealed in the composite in strong magnetic fields, which was too observed for r-GO at *T* = 300 K and polystyrene as individual components at all temperatures.

**Figure 9:**
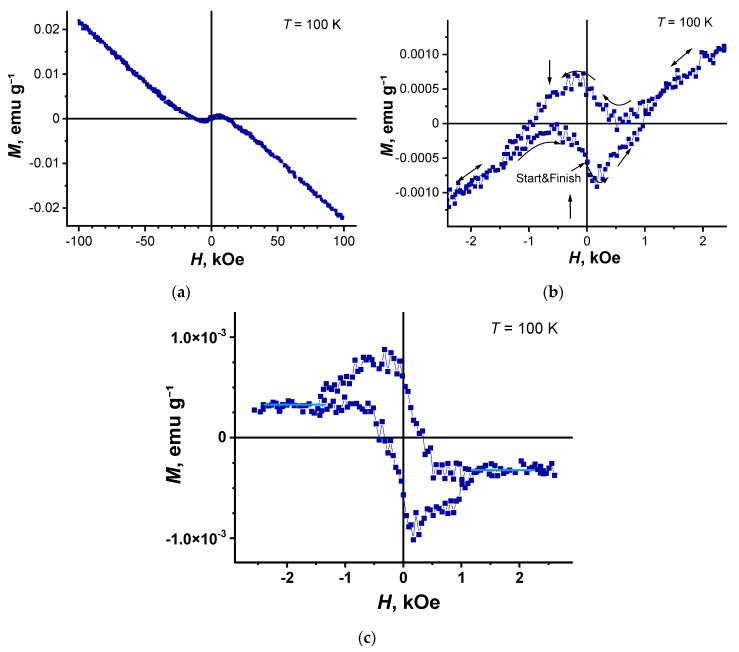
Dependence of the magnetic moment for composite 2 at *T* = 100 K on the strong (**a**) and weak magnetic fields (**b**). Arrows indicate the peaks of the magnetization loop and field direction (**b**). Magnetization loop after subtracting the diamagnetic and paramagnetic contributions (**c**).

It should be noted that this behavior, as well as a slight hysteresis observed at high temperatures in composite 2, are typical of all powder samples of granular high-temperature superconductor (HTSC) [52,53,54] and nanowire networks [55]. With decreasing temperature, the paramagnetic contribution to the total magnetization increases (Figure 6, curve 2). In this case, the form of the dependence *M*(*H*) also changes significantly (Figure 9). The magnetization presented in this figure consists of a superposition of the paramagnetic and diamagnetic contributions. As shown in Figure 9a, at *T* = 100 K in strong magnetic fields, the diamagnetic magnetizations measured at different polarities of magnetic fields are shifted relative to each other due to paramagnetic contributions. Besides, in the region of paramagnetism, a hysteresis magnetization loop is observed at weak magnetic fields at *T* = 100 K. (Figure 9b). The hysteresis curve of the magnetization of composite 2 in Figure 9b has the peaks in the weak field’s negative/positive regions, which are typical for many hard type-II superconductors [56,57].

This behavior of hysteresis is not observed in ferromagnets but is characteristic of granular type-II superconductors [52,53]. The superconducting behavior of the type-II magnetization is more clearly seen after subtracting the diamagnetic and paramagnetic contributions from the total magnetization (Figure 9c).

**Figure 10:**
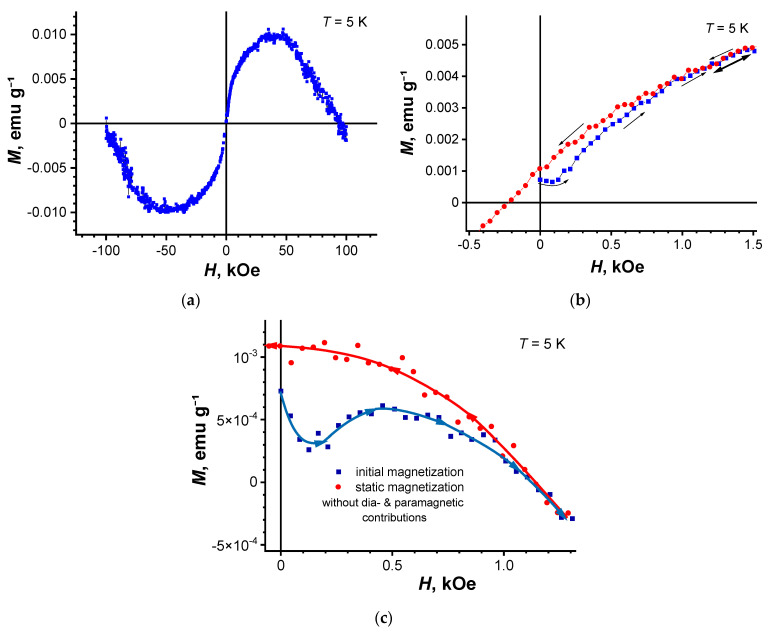
Dependence of the magnetic moment for composite 2 on the strong (**a**) and weak magnetic fields (**b**). Magnetization loop after subtracting the diamagnetic and paramagnetic contributions (**c**). *T* = 5 K. Black points mark the initial magnetization curve.

Figure 10a shows the field dependence of the static magnetization for the composite in strong magnetic fields at *T* = 5 K. As seen from Figure 10a, the magnetization is dominated by the paramagnetic effect.

Figure 10b also shows the paramagnetic signal has a hysteresis loop in weak fields (the beginning of the hysteresis loop is presented), which can be mistakenly interpreted as ferromagnetism. However, after subtracting the diamagnetic and paramagnetic contributions from the total magnetization, one can see the type-II magnetization’s superconducting behavior.

### 3.6. Discussion

As is known from the classical theory of superconductivity, penetration of a magnetic field into a type-II superconductor occurs in the form of quantized Abrikosov vortex filaments. At that, each filament has a non-superconducting core with a radius of the order of the superconducting coherence length. Around this normal-conducting cylinder, within a region with a radius of the order of the penetration depth of the magnetic field, an undamped vortex current of Cooper pairs flows. The type-II superconductor has an intermediate phase of mixed normal and superconducting properties. Vortices are fixed mainly by defects with suppressed superconductivity (pinning centers). When the external magnetic field decreases to zero, not all vortices in the sample disappear; some remain trapped at the pinning centers. In this case, the static magnetization exhibits a hysteresis loop.

Within the framework of the classical BCS superconductivity theory in conventional superconductors, the critical temperature *T*_c_ depends exponentially on the electronic density of states (DOS) at the Fermi level. In graphene, the valence and conducting bands’ structure is conical, i.e., the DOS at the Fermi level is zero; therefore, *T*_c_ should be zero also. Additionally, as follows from the BCS theory, *T*_c_ should be higher in those materials where the electron–phonon coupling strength is greater. For this reason, natural carbon is unpromising as a superconductor since this coupling is well known to be weak in carbon. Moreover, the superconducting critical temperature cannot be higher than 23 K under normal conditions in the standard BCS theory framework. Therefore, to understand superconductivity at room temperature, new theoretical scenarios must be created [58].

According to some theoretical models, *T*_c_ is predicted to range up to room temperature if the DOS is reconstructed, forming so-called flat bands in graphene [59,60,61]. It is supposed that such a DOS reconstruction will occur if graphene, multilayer graphene, or carbon flakes are subjected to mechanical stress [62,63,64,65].

For composite 2 under investigation, mechanical deformations resulting from binding organosilicon modifier TMSPM to O-containing moieties on the graphene surface led to displacement and twisting of layers in r-GO flakes. In other words, one deals with the formation of so-called twisted multilayer doped graphene. The latter is currently considered to provide the possible HTSC mechanism [5,22].

During in situ radical copolymerization of vinyl groups of styrene and TMSPM modifier on the r-GO flakes’ surface, additional chemical bonds are formed, increasing the mechanical stress inside multilayer graphene sheets.

As our studies have shown, in composite 1, in which r-GO in the dependence *M*(*T*) (see Figure 6) did not exhibit paramagnetic growth at low temperatures, O-containing moieties were few. To that point, the superconducting type of magnetization was absent in the composite because of the inability to form a sufficient number of chemical bonds between r-GO and polystyrene. It can be assumed that the superconducting form of magnetization in the composite under investigation is due to mechanical stresses in r-GO flakes. In this case, part of the r-GO flakes in the composite 2 can have strong internal stresses because of the O-containing moieties binding with the polymer. The r-GO flakes contribute to the superconducting magnetization, which appears in their temperature dependence. Indeed, the O-containing moieties that do not form chemical bonds with polystyrene via TMSPM contribute to the paramagnetism observed at low temperatures (Figure 6). So, there is considerable evidence favoring a non-significant amount of chemical binding between r-GO and polystyrene via TMSPM in composite 1 because of the small number of O-containing residues that facilitate it.

Depending on the temperature, the balance between the contributions to the total magnetization will be different. For example, at room temperature, the magnetization can be dominated by r-GO flakes with substantial deformation, which affect superconductivity. With a decrease in temperature and an increase in the fraction of magnetization from r-GO flakes with a paramagnetic contribution, the total composite magnetization should change (Figure 9 and Figure 10). The appearance of a long-range magnetic order between localized magnetic moments can undergo suppressing superconductivity in such composites. However, to conclude this section, we note that the hysteresis loop cannot be explained by a long-range ferromagnetic order since the sizes of the r-GO flakes in the composite under investigation are not few-layer-graphene nanoribbons (Figure 5), which would promote its appearance [66].

## 4. Conclusions

Synthesis of organic-inorganic composites reveals unique properties of r-GO compounds based on the two-dimensional carbon allotrope, including the relationship of magnetic with superconductive. The magnetic and conductive properties of carbon allotropes are affected by chemical dopants and chemical modification of r-GO nanosheets. The transition from native graphite to reduced graphene oxide makes it possible to achieve the desired surface modification. The latter allows the covalent binding of the r-GO flakes to the macromolecules. In such a composite, mechanical strains are possible between the layers in graphite nanosheets, which may be responsible for superconductivity. Chemists can continue the macromolecular design of composites having various deformation parameters of r-GO nanosheets, with probably room-temperature superconductivity occurring under the scenario suggested by the above theoretical works. A future challenge for chemists is to create a percolation network with similar nanographite nodes, with the organic–inorganic material retaining superconducting properties at the macrolevel.

## Figures and Tables

**Table 1:** EDX analysis data of polystyrene (PS)/laser-reduced graphene oxide (r-GO) composites and their components.

System	Composite	Elemental Composition			
		C	O	Si	S
		at.%			
r-GO (Ar)		88.16	11.84	–	–
r-GO (H_2_)		85.26	14.64	–	0.10
r-GO modified by TMSPM (Ar)		84.09	15.53	0.38	–
r-GO modified by TMSPM (H_2_)		84.93	13.96	1.08	0.03
PS		97.07	2.93	–	–
PS with r-GO modified by TMSPM (Ar)	1	96.03	3.97	–	–
PS with r-GO modified by TMSPM (H_2_)	2	97.83	2.12	0.05	–

**Table 2:** XRD analysis data of initial and surface modified r-GO flakes using Scherrer’s equation ^(1)^.

Sample	2*θ* ^(2)^, °	FWHM, °	*D_p_*^(3)^, nm	*D*^(4)^, nm	*n* ^(5)^
r-GO	24.3	2.8	2.9	0.37	8 ± 1
r-GO modified by TMSPM	24.5	2.4	3.4	0.36	9 ± 1

Note: ^(1)^ the constant in Scherrer’s equation is equal to 0.9; X-ray scattering wavelength for Cu*K*α is used: *λ* = 0.15418 nm; ^(2)^ the diffraction angle for X-ray intensity peak maximum; ^(3)^ *D_p_* is the average crystalline size; ^(4)^ *d* is the interplanar distance; ^(5)^ *n* is the number of graphene sheets in an r-GO flake.

## Data Availability

Data is contained within the article or supplementary material. Besides, the data presented are available on request from the corresponding author.

## Supplementary Materials

The following are available online at https://www.mdpi.com/2079-4991/11/2/403/s1, Figure S1: Scheme of a composite based on polystyrene/reduced graphene oxide, Figure S2: EDX spectra of composite 2 and their components: PS with r-GO modified by TMSPM (1), PS (2), r-GO (3), r-GO modified with TMSPM (4), Figure S3: Raman spectra for the r-GO obtained in an Ar (**a**) and H_2_ (**b**) atmosphere, Figure S4: Raman spectrum for the r-GO obtained in an H_2_ atmosphere. An example of five functions (D″, D, D*, G, and M bands) deconvolution for the r-GO obtained (**a**). The summed peaks in the spectrum are marked (**b**), Table S1: The position of different modes in cm^−1^ in the Raman spectrum for r-GO obtained in an H_2_ atmosphere.
