# Supplementary material for: The Magnetization of a Composite Based on Reduced Graphene Oxide and Polystyrene
Source: Nanomaterials (Basel). 2021 Feb 5;11(2):403. doi: 10.3390/nano11020403 (PMC7915180; doi:10.3390/nano11020403)
Supplement: Supplementary file 1 [file nanomaterials-11-00403-s001.pdf]

nanomaterials

MDPI

Article

Electronic Supplementary Materials: **The magnetization of a composite based on reduced graphene oxide and polystyrene**

Alexander N. Ionov[1], Mikhail P. Volkov[1], Marianna N. Nikolaeva[2], Ruslan Y. Smyslov[2,3], and Alexander N. Bugrov[2,4,*]

[1] Ioffe Institute, Politekhnicheskaya 26, St. Petersburg 194021, Russian Federation; ionov@tuch.ioffe.ru (A.N.I.); m.volkov@mail.ioffe.ru (M.P.V.)
[2] Institute of Macromolecular Compounds, Russian Academy of Sciences, Bolshoy pr-t 31, St. Petersburg 199004, Russian Federation; marianna_n@mail.ru (M.N.N.); urs@macro.ru (R.Y.S.); anbugrov@etu.ru (A.N.B.)
[3] Peter the Great St.Petersburg Polytechnic University (SPbPU), Institute of Biomedical Systems and Biotechnology, Graduate School of Biomedical Systems and Technology, Polytechnicheskaya 29, St.Petersburg 195251, Russian Federation
[4] Saint Petersburg Electrotechnical University (ETU "LETI"), Department of Physical Chemistry, ul. Professora Popova 5, St. Petersburg 197376, Russian Federation
* Correspondence: anbugrov@etu.ru; Tel.: +7-812-323-6269

1. Introduction

As far as materials science is concerned, our work aims to comprehend the functionalization of reduced graphene oxide (r-GO). Understanding the graphite nanosheet surface and how it can be engineered through macromolecular design can be crucial in many relevant fields: photovoltaics, catalysis, fuel cells, sensors, and batteries. [S1].

2. Materials, Methods, and Results

IR spectroscopy was used to characterize the surface of the used r-GO nanosheets chemically. The Raman spectroscopy allowed us to describe the differences in the defect structure of the r-GO nanosheets under study. XRD made it possible to study the crystal structure and the degree of their dispersion. As the dispersed phase in the composition of polystyrene-based composites were chosen r-GO, obtained by heat treatment of graphite oxide in argon (900 °C) and molecular hydrogen (900 °C) (Table 1 in Article). For further synthesizing composites 1 and 2, one used r-GO upon reducing in an argon or hydrogen atmosphere, correspondingly (Table 1 in Article).

2.1. Functionalization and synthesis

In Figure S1. we have presented the possible scheme for covalent bonding between r-GO nanosheets and polystyrene macromolecules via 3-(trimethoxysilyl) propyl methacrylate (TMSPM) binding.

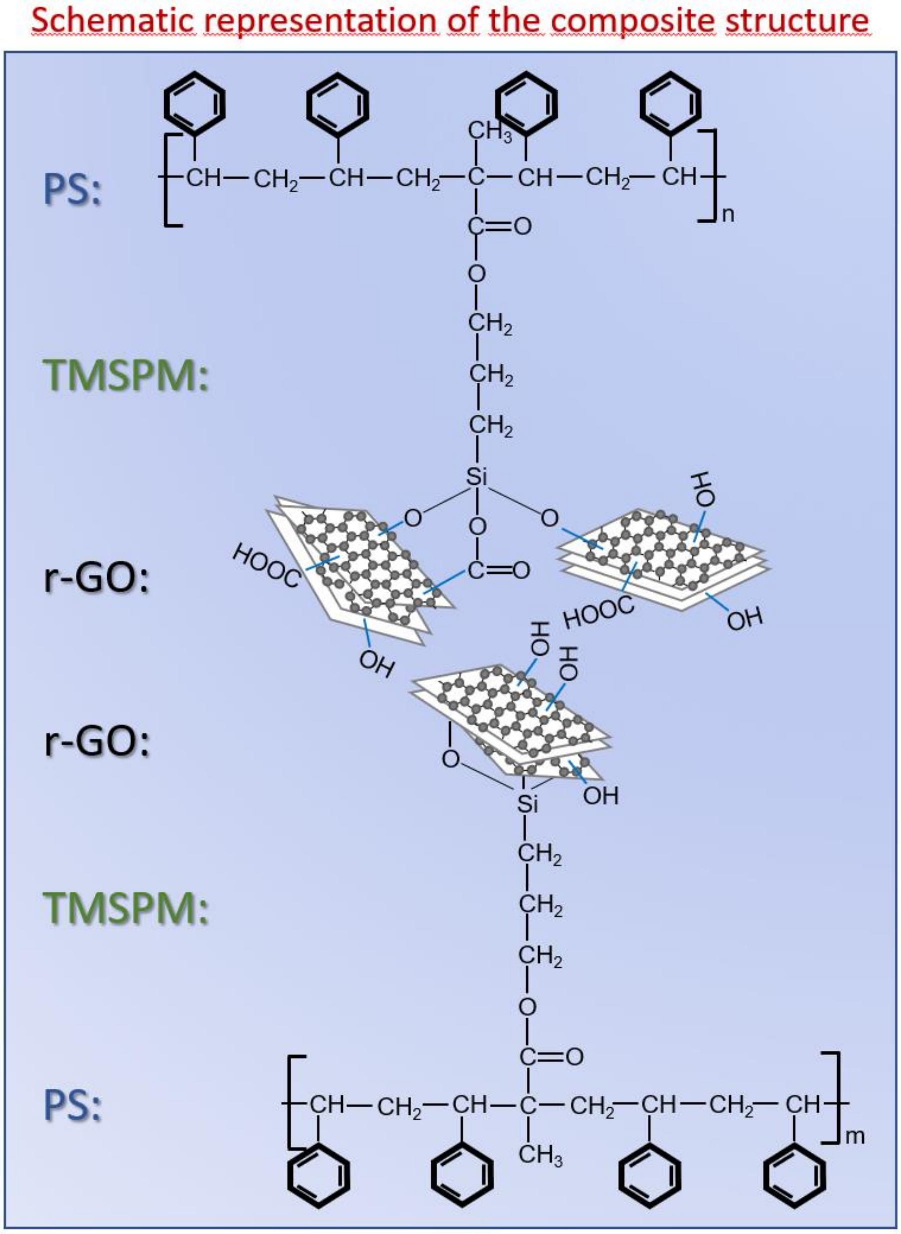

Figure S1. Scheme of a composite based on polystyrene/reduced graphene oxide.

2.2. Impurity control

The chemical compositions were determined from the maximum possible area of the test samples. EDX analysis confirms the absence of 3d metal impurities in native or TMSPM modified r-GO flakes and those included in the composite structure (Table 1 in Article). In this case, the presence of an organosilicon compound responsible for the covalent binding of r-GO flakes to styrene macromolecules can be traced in the EDX spectra for composite 2 (Figure S2).

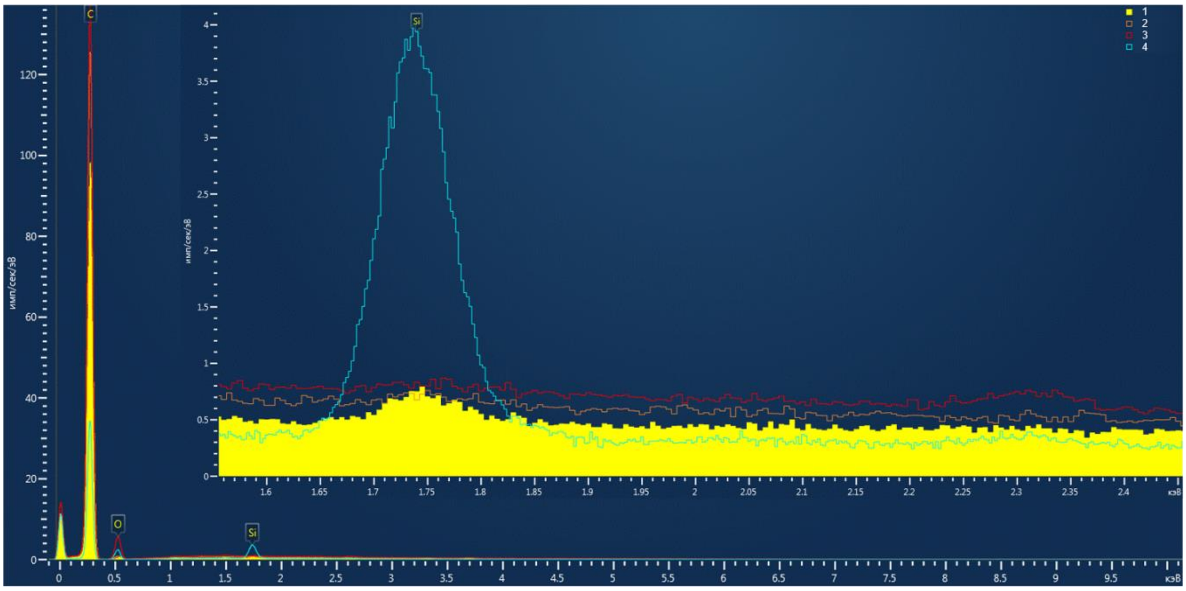

Figure S2. EDX spectra of composite 2 and their components: PS with r-GO modified by TMSPM (1), PS (2), r-GO (3), r-GO modified with TMSPM (4).

2.3. Raman analysis of initial and surface modified r-GO flakes

Based on the model-based notions about the nature of the peaks [S2] on the Raman curves, we deconvolved the spectral contour using the Voigt function in the OriginPro 2021 software. This approach is close to that described in [S3]. Figure S3 shows the deconvolution of the spectra for r-GO treated in an Ar and H_2 atmosphere. In these decompositions, only the single parameter y0 was fixed, which corresponds to the subtraction of baseline.

Raman shifts less than 1000 cm^{-1} were not considered since they can be associated with artifacts arising during sample preparation. This range is also challenging to interpret due to the lack of reliable data reviewed in the scientific literature.

Table S1. The position of different modes in cm^{-1} in the Raman spectrum for r-GO obtained in a H_2 atmosphere. See Figure S3 and S4.

Mode	Itself	+ D"	+ D	+ D*	+ M
D"	1150	–	2502	–	–
D	1352	2502	2704	2898	3112
D*	1546	–	2898	–	–
G	1603	2753	2955	–	–
D'	1615 [1)]	–	2967	–	–
M	1760	–	3112	–	–

Note: [1)] Difficult to deconvolute from the spectrum obtained.

In Table S1, we have tried to identify the summed peaks observed in the region of Raman shifts 2300–3300 cm^{-1} for doubled modes. The complex peak of 2850–3000 cm^{-1} may be associated with the sum of the main mode D and some others.

According to Vollebregt et al. [S4], the D* band is related to amorphous carbon phases.

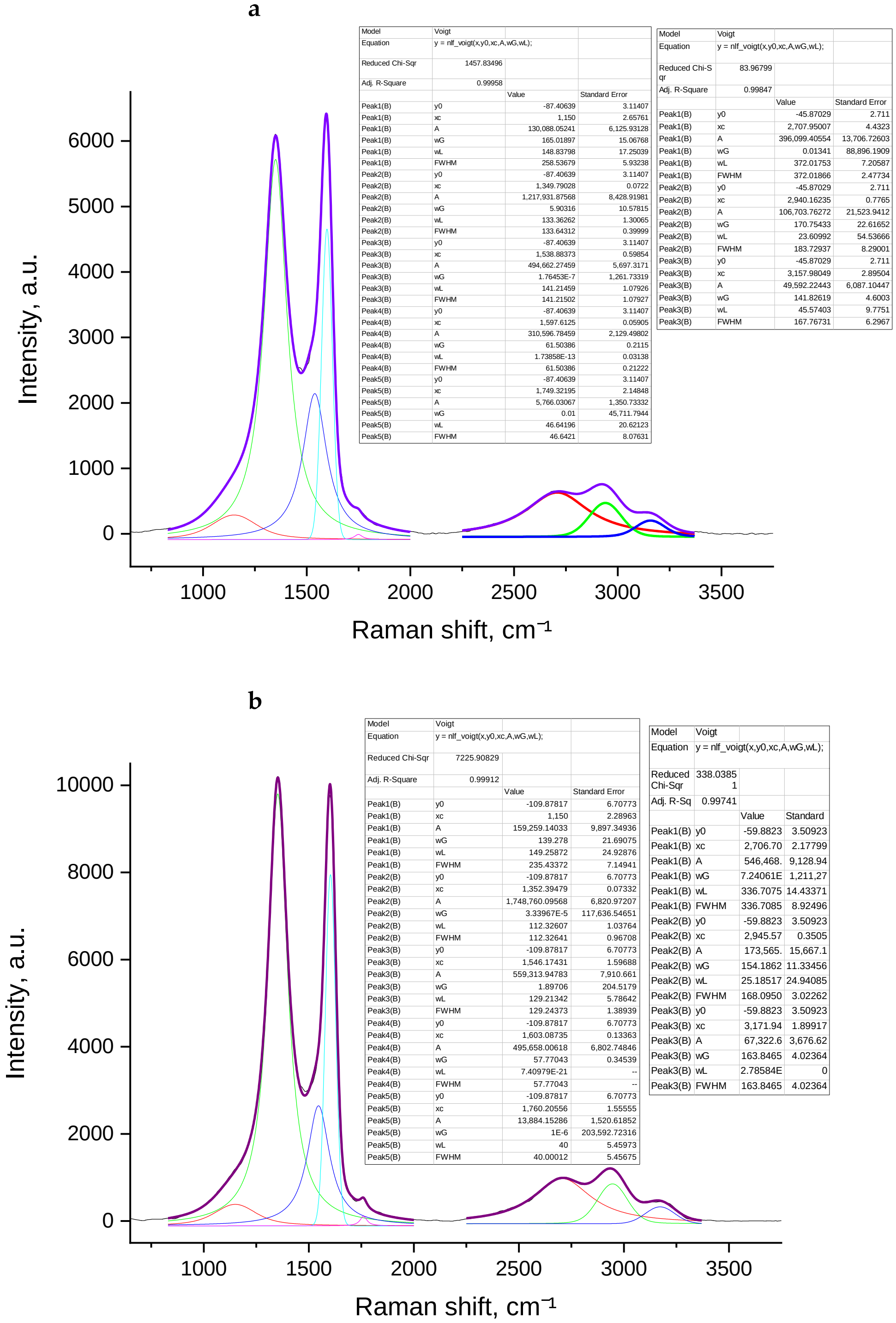

Figure S3. Raman spectra for the r-GO obtained in an Ar (**a**) and H_2 (**b**) atmosphere.

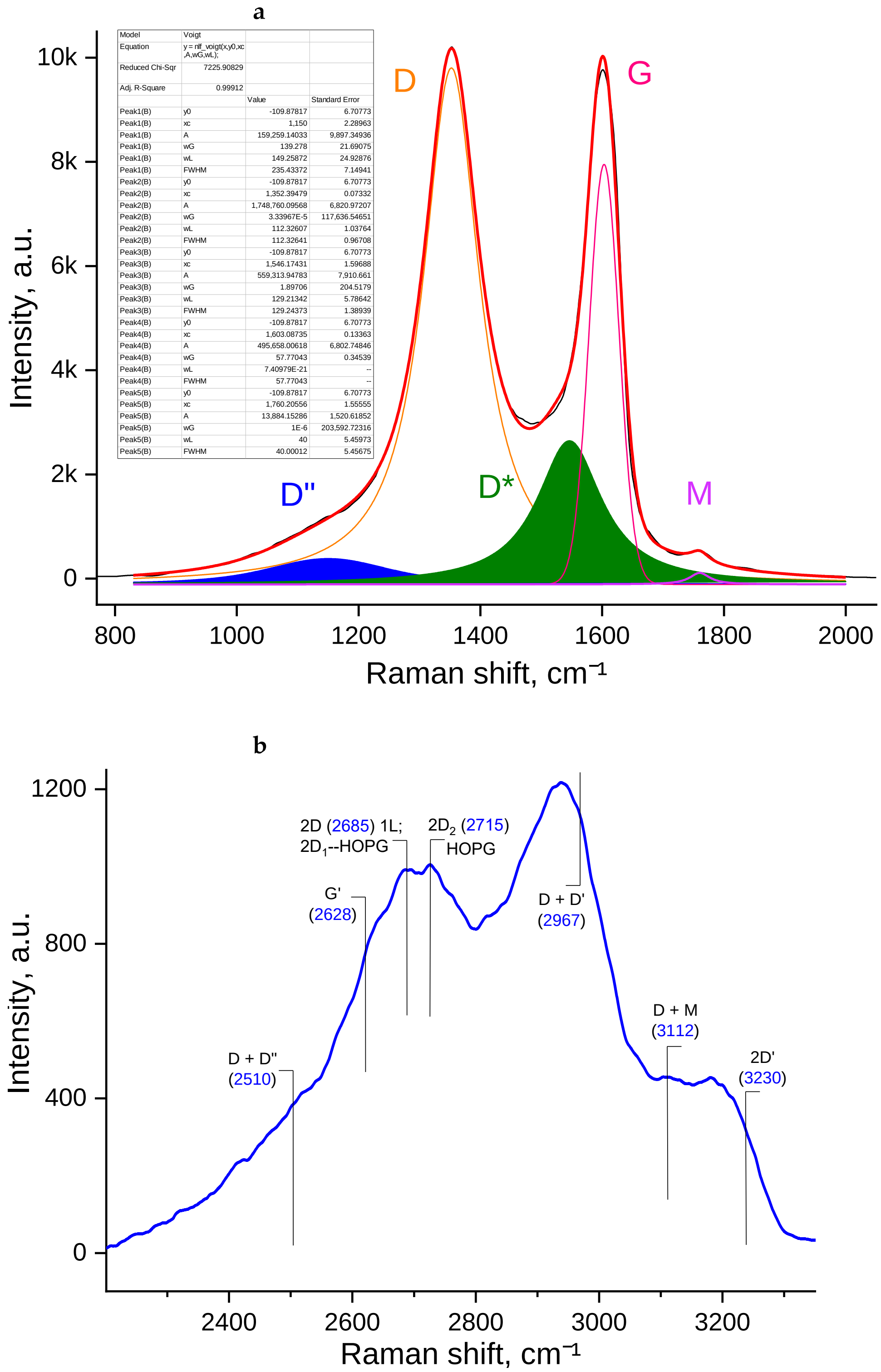

Figure S4. Raman spectrum for the r-GO obtained in a H_2 atmosphere. An example of five functions (D″, D, D*, G, and M bands) deconvolution for the r-GO obtained (**a**). The summed peaks in the spectrum are marked (**b**).

References

S1. Kuila, T.; Bose, S.; Mishra, A.K.; Khanra, P.; Kim, N.H.; Lee, J.H. Chemical Functionalization of Graphene and Its Applications. *Progress in Materials Science* **2012**, *57*, doi:10.1016/j.pmatsci.2012.03.002.

S2. Ferrari, A.C.; Basko, D.M. Raman Spectroscopy as a Versatile Tool for Studying the Properties of Graphene. *Nature Nanotechnology* **2013**, *8*, doi:10.1038/nnano.2013.46.

S3. Ma, B.; Rodriguez, R.D.; Ruban, A.; Pavlov, S.; Sheremet, E. The Correlation between Electrical Conductivity and Second-Order Raman Modes of Laser-Reduced Graphene Oxide. *Physical Chemistry Chemical Physics* **2019**, *21*, doi:10.1039/C9CP00093C.

S4. Vollebregt, S.; Ishihara, R.; Tichelaar, F.D.; Hou, Y.; Beenakker, C.I.M. Influence of the Growth Temperature on the First and Second-Order Raman Band Ratios and Widths of Carbon Nanotubes and Fibers. *Carbon* **2012**, *50*, doi:10.1016/j.carbon.2012.03.026.
